# Transcription Factors Are Involved in Wizened Bud Occurrence During the Growing Season in the *Pyrus pyrifolia* Cultivar ‘Sucui 1’

**DOI:** 10.3390/epigenomes8040040

**Published:** 2024-10-25

**Authors:** Hui Li, Jialiang Kan, Chunxiao Liu, Qingsong Yang, Jing Lin, Xiaogang Li

**Affiliations:** 1 Institute of Pomology/Jiangsu Key Laboratory for Horticultural Crop Genetic Improvement, Jiangsu Academy of Agricultural Sciences, Nanjing 210014, China; 20010005@jaas.ac.cn (H.L.);; 2 State Key Laboratory of Subtropical Silviculture, Zhejiang A&F University, Lin’an 311300, China

**Keywords:** pear, wizened bud, transcription factor, gene expression, methylation feature

## Abstract

Background: Flowers are important plant organs, and their development is correlated with yield in woody fruit trees. For *Pyrus pyrifolia* cultivar ‘Sucui 1’, the research on how DNA methylation accurately regulates the expression of TFs and affects the specific regulatory mechanism of flower bud wizening will help reduce wizened buds. Methods: Here, the DNA methylomes and transcriptomes of two types of flower buds from the *Pyrus pyrifolia* cultivar ‘Sucui 1’ were compared. Results: 320 differentially expressed transcription factors (TFs), in 43 families, were obtained from the wizened bud transcriptome versus the normal bud transcriptome. Most were members of the AP2/ERF, bHLH, C2H2, CO-like, MADS, MYB, and WRKY families, which are involved in flower development. As a whole, the methylation level of TFs in the ‘Sucui 1’ genome increased once flower bud wizening occurred. A cytosine methylation analysis revealed that the methylation levels of the same gene regions in TFs from two kinds of buds were similar. However, differentially methylated regions were found in gene promoter sequences. The combined whole-genome bisulfite sequencing and RNA-Seq analyses revealed 162 TF genes (including 164 differentially methylated regions) with both differential expression and methylation differences between the two flower bud types. Among them, 126 were classified as ^m^CHH-type methylation genes. Furthermore, the transcriptional down regulation of *PpbHLH40*, *PpERF4*, *PpERF061*, *PpLHW*, *PpMADS6*, *PpZF-HD11*, and *PpZFP90* was accompanied by increased DNA methylation. However, *PpbHLH130*, *PpERF011*, and *PpMYB308* displayed the opposite trend. The expression changes for these TFs were negatively correlated with their methylation states. Conclusions: Overall, our results offer initial experimental evidence of a correlation between DNA methylation and TF transcription in *P. pyrifolia* in response to bud wizening. This enriched our understanding of epigenetic modulations in woody trees during flower development.

## 1. Introduction

Pear (*Pyrus* spp.) is a traditional economic crop, ranks third among temperate fruits, and is popularly planted worldwide [1]. This species is a perennial plant, belonging to the genus *Pyrus*, family Rosaceae. Its yield is affected by many factors, especially the differentiation and development of flower buds. In the field, the quality of pear flower buds is closely related to the branches’ growth conditions [2]. If adverse temperature or air (humidity) conditions occur during the developmental process, then the external scales of flower buds become loose and the internal organs turn brown. They are then transformed to non-functional and wizened flower buds (SM) [3]. If such unfavorable conditions occur during spring, flower buds may have difficulty germinating, leading to SM that drop from branches [4]. Thus, the SM phenomenon is a serious threat to pear production.

The formation of SM is commonly observed in many varieties of pear trees [3,4,5], resulting in considerable decreases in fruit yield. Usually, the differences in physiology and transcriptomics occur in relation to the formation of SM [3]. Indeed, transcriptional changes in serial genes have been detected in SM. For example, transcription factor (TF) *PpyMYB39.1* reduces in expression level and may play a vital role in SM formation [3]. Additionally, DNA methylation plays a part in the plant developmental processes by manipulating gene expression [6,7]. For instance, the regulation of gene expression through DNA methylation influences apple flower development and formation [8]. Moreover, stress-regulated flowering coordinates gene transcription patterns through epigenetic-based DNA methylation [9]. However, the intrinsic molecular mechanisms related to SM formation in pears remain vague. Additionally, the association of DNA methylation with gene transcription needs to be explored to understand its role in SM formation.

Plant flower development is modulated by TFs, because they manipulate floral traits by controlling the expression levels of multiple genes [10]. First, the famous floral MADS-box TFs link organ specification, growth, and cell differentiation [11]. These genes control floral organ identities by forming particular complexes, and their combination patterns are conserved among diverse plants [12]. *Pyrus pyrifolia AP1*, a MADS-box gene in pears, plays important roles in regulating vegetative to reproductive development and the development of floral organs [13]. Other TFs are also involved in regulating plant reproductive processes. For example, *AP2/ERF* genes convert leaves into floral structures [14], which regulate spikelet determinacy, floral organ development, and flowering time in rice [15]. MYB and bHLH families positively control the floral transition [14], and members of the former often form regulatory complexes with members of the latter. Like other plants, *PyMYB*s interact with bHLHs in red sand pears [16]. Additionally, MYB takes part in male reproductive development at three stages, including the formation and maturation of pollen grains [17]. However, whether these TFs are involved in SM formation is still unknown. The identification of differentially expressed TFs by comparing the transcriptome of SM and normal flower buds (CKM) is helpful in fully understanding their roles during this process.

The sand pear (*P. pyrifolia*) variety ‘Sucui 1’ is an offspring of ‘Huasu’ (♂) × ‘Cuiguan’ (♀) created in 2011 that matures at the end of June. However, SM is often observed in this variety if the temperature rises abnormally or rainfall decreases in autumn. This phenomenon reduces the number of CKM, affecting the next year’s yield. Here, we investigated this process. First, the transcriptome and methylome profiles of SM and CKM were compared, and their differentially expressed transcription factors (DTFs) were explored. Then, the expression levels and methylation states of DTFs from the two kinds of flower buds were analyzed. Finally, the possible mechanisms underlying SM formation that are regulated by TFs were investigated. The results provide new insights into reducing or even eliminating SM occurrence in pear trees.

## 2. Results

### 2.1. Transcriptionally Differential TFs from Two Types of Pear Flower Buds

**Figure 1:**
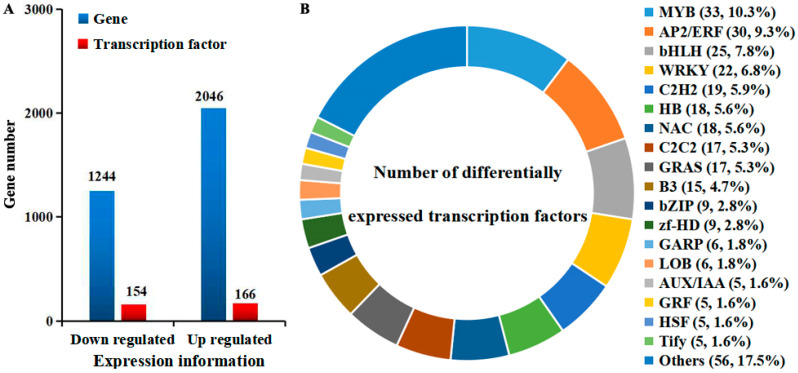
Numbers of differentially expressed genes (DEGs) (**A**) and types and proportions of differentially expressed transcription factors (**B**) in normal flower buds versus wizened flower buds of *Pyrus pyrifolia* cultivar ‘Sucui 1’, as determined by a transcriptome analysis. The number indicates the number of differentially expressed transcription factor genes and the percentage represents the proportion of the family members of the total differentially expressed transcription factors. A list of abbreviations is given in Supplementary Materials, Table S3.

**Figure 2:**
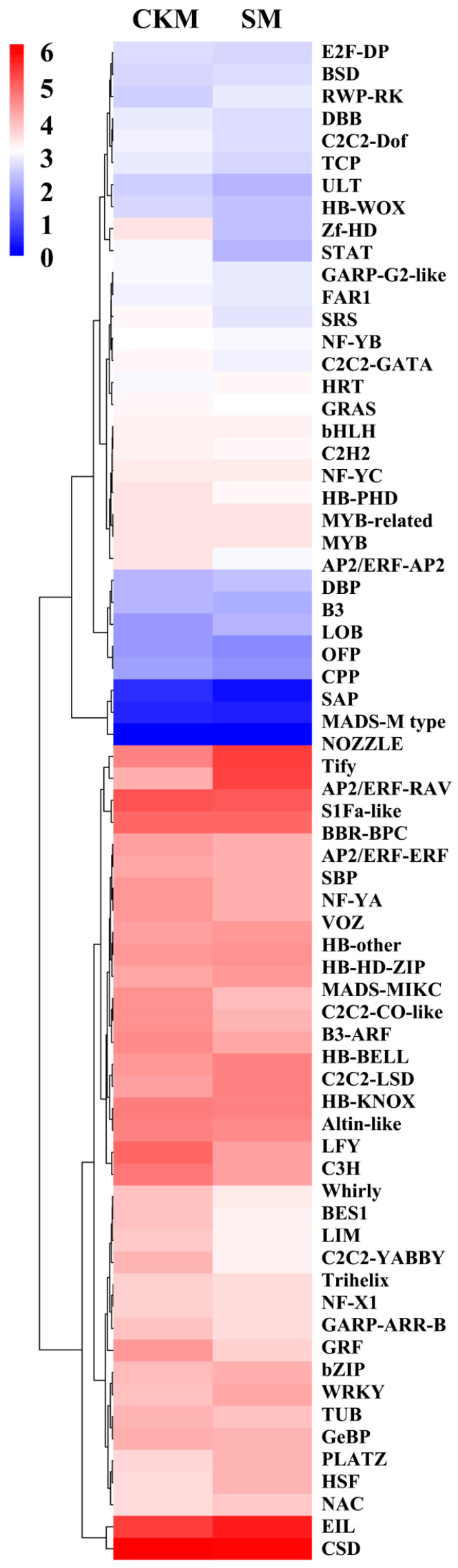
The heat maps of transcription factor expression in different flower buds of *Pyrus pyrifolia* cultivar ‘Sucui 1’ based on transcriptome sequencing results. CKM, normal flower buds; SM, wizened flower buds. A list of abbreviations is given in Supplementary Materials, Table S3.

A total of 25.8–28.1 million valid transcriptome sequences were obtained from six ‘Sucui 1’ pear flower bud samples, with Q30 ≥ 90.5% and ≥ 7.7 Gb data per sample. A total of 48.4 Gb data were obtained for subsequent analyses (NCBI number: PRJNA951885). The consistency among the three biological replicates for each treatment was 89.2–96.2%. The similarity between the transcriptome data and the pear reference genome (http://ngdc.cncb.ac.cn/gwh/Assembly/647/show, accessed on 6 March 2023) was 81.6–86.7%. With the help of iTAK (v1.7a) software, 3335 TF genes were identified from the transcriptome data of ‘Sucui 1’ pear flower buds (Figure 1A). In addition, these TFs were distributed among 94 families. The transcriptome data of CKM and SM in the ‘Sucui 1’ pear were compared and analyzed, and 320 DTFs (166 up regulation genes and 154 down regulation genes, Figure 1B) were obtained. The functions of the DEGs were investigated through a KEGG pathway analysis and GO functional annotation (Supplementary Materials, Tables S1 and S2). These DTFs were distributed across 43 families, including AP2/ERF, bHLH, C2H2, CO-like, MADS, MYB, and WRKY (Figure 1B, Table 1), whose members are closely associated with flower development. Among them, the numbers of DEGs in the MYB, AP2/ERF, and bHLH families were the highest, at 33, 30, and 25, respectively. Furthermore, the proportions of genes showing differential expression from zf-HD (36%), GRF (31.3%), and SNF2 (28.6%) were the greatest, which indicated that each family had differentially regulated rules once wizened buds appeared. The expression heat maps of the TFs are shown in Figure 2.

### 2.2. Differential Methylation Regions in TFs of the Two Types of Pear Flower Buds

**Figure 3:**
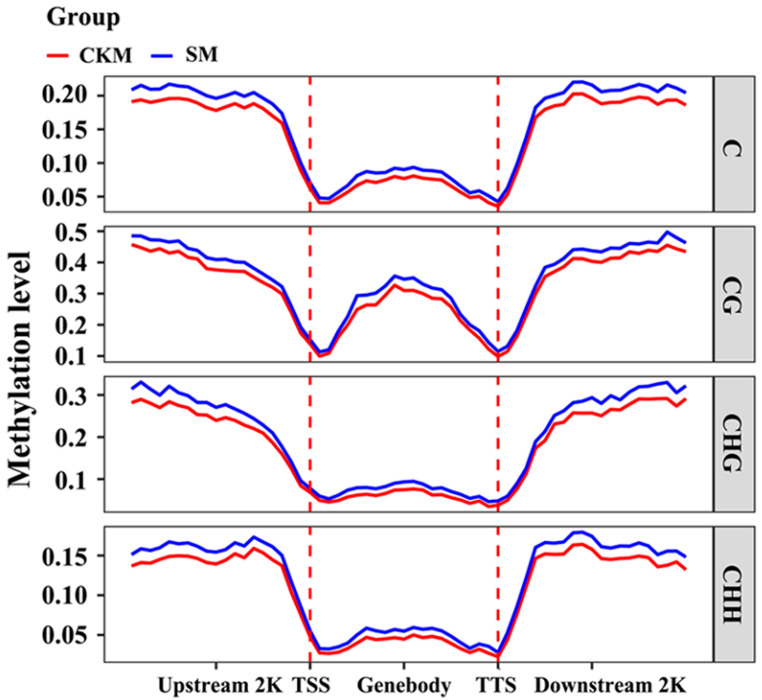
Distribution of methylation levels in different gene regions of transcription factors in flower buds of *Pyrus pyrifolia* cultivar ‘Sucui 1’ based on the whole-genome bisulfite sequencing result. CKM, normal flower buds; SM, wizened flower buds; 2K, 2 kilobase; TSS, transcription initiation site; TTS, transcription termination site.

The WGBS raw data of 6 ‘Sucui 1’ pear flower bud samples were filtered to obtain 62.5–91.8 million effective sequences, with Q30 ≥ 89.2% and 17.5–25.7 Gb data (NCBI number: PRJNA951883). The WGBS data were 36.1–48.2 times the pear reference genome size (532.7 Mb, https://www.ncbi.nlm.nih.gov/datasets/genome/GCA_007844245.1/, accessed on 6 March 2023) [18]. The coverage of the sample WGBS data in this genome was 71.4–74.1% (covering 1× of the genome). The methylation levels of the same TF gene regions from the two samples were similar (Figure 3). First, the transcription initiation and transcription termination sites were set as the upstream and downstream boundaries, respectively, of all the genes. For example, the methylation level of the gene coding region was the lowest, whereas the methylation levels of the gene upstream and downstream regions (2 kb each) were the highest. After flower buds wizened, the methylation level of TFs in the ‘Sucui 1’ genome increased and the regions of differential methylation between CKM and SM occurred mainly in gene promoter sequences (Figure 3).

**Figure 4:**
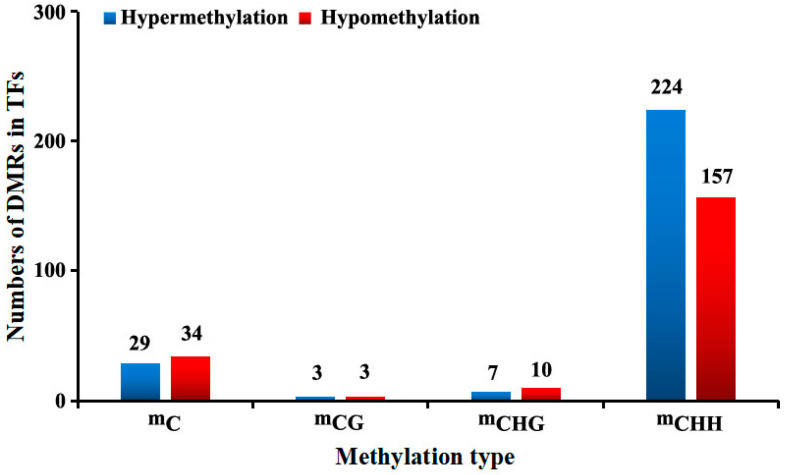
The numbers of differentially methylated regions (DMRs) in two flower buds of the *Pyrus pyrifolia* cultivar ‘Sucui 1’ based on the whole-genome bisulfite sequencing results. TFs, transcription factors.

**Figure 5:**
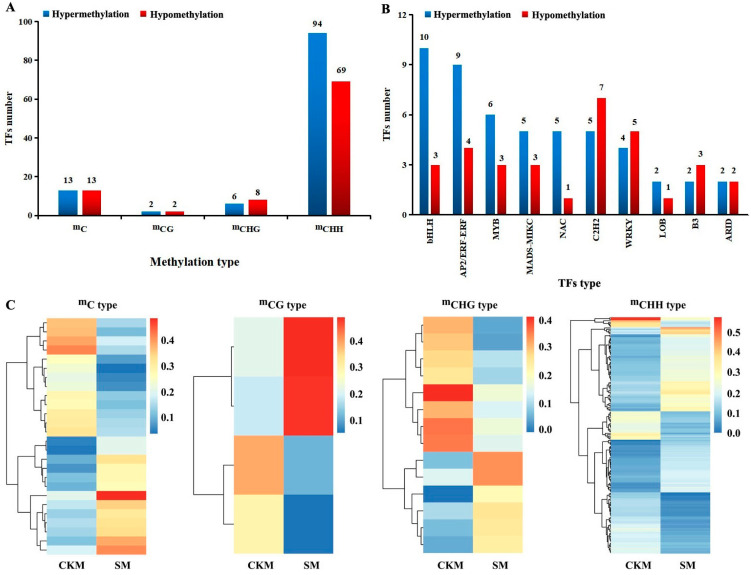
The number of differentially methylated transcription factors (TFs, (**A**)), their family distribution (^m^CHH type, (**B**)) and the heat maps of DMRs in DMGs from TFs (**C**) in different buds of the *Pyrus pyrifolia* cultivar ‘Sucui 1’ based on the whole-genome bisulfite sequencing results. CKM, normal flower buds; SM, wizened flower buds. A list of abbreviations is given in Supplementary Materials, Table S3.

By comparing the WGBS data from CKM and SM, 263 (hyper-methylation level) and 204 (hypo-methylation level) DMRs (including ^m^C, ^m^CG, ^m^CHG, and ^m^CHH types) in the TF genes were identified, respectively. Among them, the ^m^CHH-type DMRs accounted for 85.1% and 76.8% of the total DMRs in CKM and SM, respectively (Figure 4). The WGBS data for CKM and SM were compared, revealing 115 (methylation level increased) and 92 (methylation level decreased) DMGs from the TFs (including ^m^C, ^m^CG, ^m^CHG and ^m^CHH types), respectively. Among them, the ^m^CHH-type DMRs accounted for 81.7% (methylation level increased) and 75.0% (methylation level decreased) of the total DMRs in CKM and SM, respectively (Figure 5A). Figure 5B displays the top 10 gene families showing the most changes in CHH methylation levels in the ‘Sucui 1’ genome. The numbers of members with increased and decreased methylation levels among different TF families differed, indicating that many TFs are involved in flower bud wizening, but their specific roles vary. Moreover, the heat maps of the methylation levels of DMRs in DMGs from TFs in the two samples are shown in Figure 5C.

### 2.3. Joint Analysis and Validation of Methylation and Transcriptome Data

The combined analysis of WGBS and RNA-Seq data revealed 162 TF genes (including 164 DMRs) with both differential expression and methylation differences in ‘Sucui 1’ after flower buds wizened. Among these genes, 90 and 72 members had increased and decreased methylation levels, respectively (Table 2). Specifically, there were 21 ^m^C-type, 4 ^m^CG- or 11 ^m^CHG-type, and 126 ^m^CHH-type differentially methylated TF genes, respectively.

**Figure 6:**
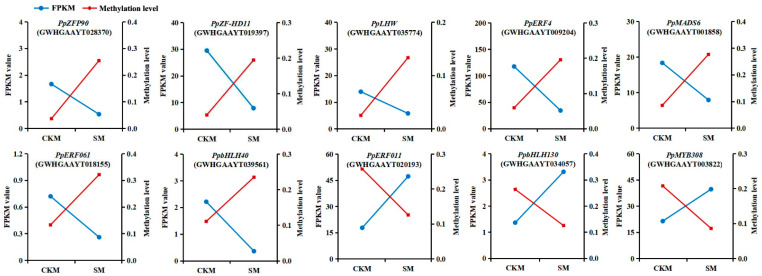
The fragments per kilo-base per million read values (FPKM valued, blue points) and DNA methylation levels of ^m^CHH-type differentially methylated regions (red points) in transcription factor genes in different buds of *Pyrus pyrifolia* cultivar ‘Sucui 1’ based on transcriptome sequencing and whole-genome bisulfite sequencing results, respectively. CKM, normal flower buds; SM, wizened flower buds.

Ten TFs with negative correlations between their gene transcription changes and DNA methylation states were selected (their specific information is given in Table 3 and Supplementary Materials, Table S4). Here, we show their ^m^CHH methylation levels and expression levels in pear flower buds after wizening occurred (Figure 6). Inverse patterns between transcripts and DNA methylation levels at the test times were observed for two *bHLH*s, three *ERF*s, and five other family members. In detail, seven genes, *PpbHLH40* (GWHGAAYT039561), *PpERF4* (GWHGAAYT009204), *PpERF061* (GWHGAAYT018155), *PpLHW* (GWHGAAYT035774), *PpMADS6* (GWHGAAYT001858), *PpZF-HD11* (GWHGAAYT019397), and *PpZFP90* (GWHGAAYT028370), with hypermethylation in the ^m^CHH context, showed decreased transcription levels. Meanwhile, *PpbHLH130* (GWHGAAYT034057), *PpERF011* (GWHGAAYT020193), and *PpMYB308* (GWHGAAYT003822) showed hypomethylation in the ^m^CHH context and showed increased transcription levels. In addition, the above methylation changes occurred in the promoter regions or in the downstream regions after the gene transcription terminator.

**Figure 7:**
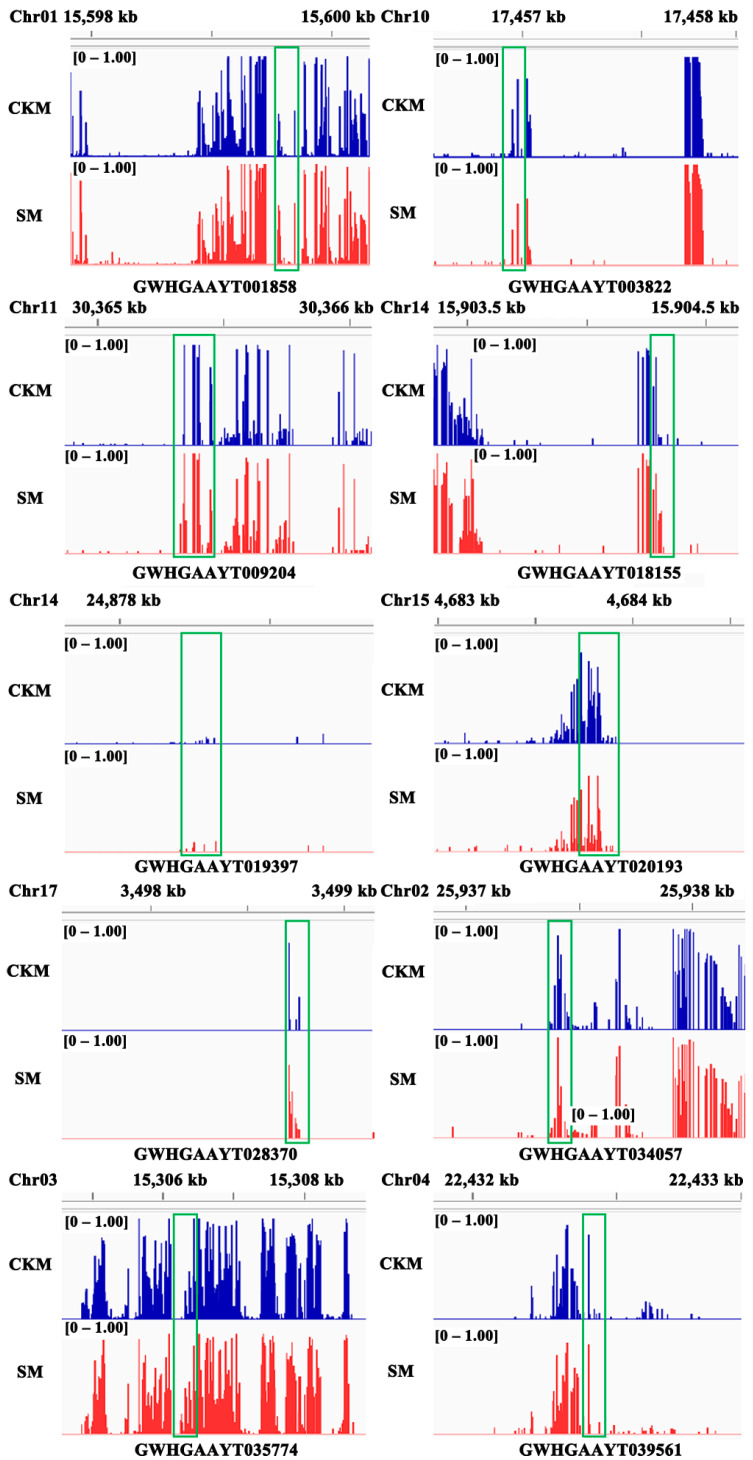
IGV software depiction of the methylation states of differentially methylated regions of the 10 genes in wizened flower buds (SM) versus normal flower buds (CKM) of the *Pyrus pyrifolia* cultivar ‘Sucui 1’ as assessed by whole-genome bisulfite sequencing. DMRs are marked with green boxes; [0–1.00] indicates the methylation level range of ^m^CHH sites.

**Figure 8:**
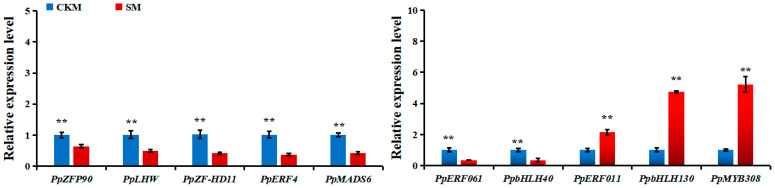
qPCR analysis of the transcription levels of differentially methylated region-associated transcription factor genes in different buds of the *Pyrus pyrifolia* cultivar ‘Sucui 1’. *PbEF-1α* was selected as an internal control gene for normalization. The experimental data were tested via SPSS 26 (IBM, Armonk, NY, USA), and values are shown as the means ± standard deviations (SDs). SDs of the means of three biological replicates are displayed as vertical bars. The significant differences (** *p* < 0.01) in gene expression data between normal flower buds (CKM) and wizened flower buds (SM) were analyzed using Student’s *t*-tests.

Furthermore, IGV software (v2.13.2) was used to identify ^m^CHH-type DMRs in the two samples (Figure 7). In total, 10 candidate TFs from the DEGs were chosen and analyzed via qPCR to verify the accuracy of the transcriptome sequencing results. Their expression levels in CKM and SM are displayed in Figure 8. For example, the expression levels of *PpbHLH40* (GWHGAAYT039561), *PpERF061* (GWHGAAYT018155), *PpERF4* (GWHGAAYT009204), *PpLHW* (GWHGAAYT035774), *PpMADS6* (GWHGAAYT001858), *PpZF-HD11* (GWHGAAYT019397), and *PpZFP90* (GWHGAAYT028370) in SM are significantly lower than in CKM. However, the expression levels of *PpbHLH130* (GWHGAAYT034057), *PpERF011* (GWHGAAYT020193), and *PpMYB308* (GWHGAAYT003822) show the opposite trend (Table 4).

To further analyze correlations between gene expression and DNA methylation in the ^m^CHH context, we tested the DNA methylation statues and transcription levels of the above 10 TFs. Table 4 shows that the expression features of these genes are basically consistent with the transcriptome sequencing results. This suggested that the results of the DEG analysis are reliable. Their transcription level changes are negatively correlated with methylation changes. In the flower buds of ‘Sucui 1’, how DNA methylation accurately controls the transcription of specific genes and affects the specific regulatory mechanisms of flower bud loosening still needs to be explored further.

## 3. Discussion

### 3.1. TFs Involved in the Wizening of Pear Flower Buds

It is a general phenomenon that gene transcription changes at the genome-wide level, as plants transition from vegetative to reproductive growth [19]. Although the sand pears ‘Sucui 1’ and ‘710’ both exhibited typical wizened buds in September, their transcription features are different. Many DEGs have been identified in ‘710’ between normal buds and wizened buds [3], but we acquired more than six-fold DEGs in ‘Sucui 1’. Among them, 6 out of 10 had up regulated expression. Additionally, KEGG pathway and GO enrichment analyses for DEGs from the pear cultivars ‘710’ and ‘Sucui 1’ revealed different features, which may be dependent on their varietal characteristics. While ‘710’ is prone to wizened buds and its fruit is brown [3], ‘Sucui 1’ has green fruit. In detail, DEGs from ‘Sucui 1’ were enriched in ‘male–female gamete recognition during double fertilization forming’, ‘photosynthetic NADP+ reduction’, ‘shoot organ boundary specification’, ‘specification of plant organ position’, and ‘leading strand elongation’. Meanwhile, ‘response to wounding’, ‘suberin biosynthetic process’, ‘regulation of defense response’, ‘syncytium formation’, ‘nitrate transport’, and ‘plant-type cell wall loosening’ processes were the top five enriched GO pathways from ‘710’ [3]. In addition, some kinds of genes showed different expression trends in two cultivars. For example, *PpMYB308* of pear ‘Sucui 1’ increased the expression amount and *PpyMYB39.1* of pear ‘710’ reduced transcript levels in wizened buds [3]. Furthermore, TFs form the hub of RNA transcriptional network regulation, controlling floral transition and flower formation [10,20]. Their altered expression levels are particularly common throughout flower bud development. For instance, the expression levels of many TFs increase or decrease in *Arabidopsis*, *Litsea cubeba*, celery, and apple during different stages of flower bud development [8,11,21,22].

Here, we focused on TF expression levels in SM in the pear variety ‘Sucui 1’ during flower development. In total, 9.5% (320 of 3335) of TF genes were up regulated or down regulated in flower buds once the wizening phenomenon occurred. We identified more than 54 TFs among the DEGs from SM of the anther sand pear variety ‘710’ [3]. First, 30 members of AP2/ERF, a large group of factors that are essential in the criterion of floral organ status, the foundation of the floral meristem, and the adjustment of gene transcription during flower development [11,23] displayed different expression levels in CKM and SM in the pear variety ‘Sucui 1’. These AP2 genes may be implicated in the formation of SM, but their specific roles need further research. Another TF family, MADS-box, acts in partly stage- and tissue-specific manners during flower development. Moreover, all the floral homeotic TFs belong to the MIKC-type class [10,24]. We found two MADS-MIKC genes that had expression changes by comparing the transcriptomes of CKM and SM. Thus, the MIKC-type class may be involved in flower abortion during abnormally high temperatures in autumn. Moreover, transcription changes for 25 MYB genes were detected in SM in ‘Sucui 1’. These genes may promote pectin degradation and reduce carbohydrate transport, like *PpyMYB39.1* in the anther sand pear variety ‘710’ [3]. However, their downstream genes and how they regulate the cell wall loosening process are still unknown. Thus, TFs appeared to play vital roles in SM formation among different kinds of pear species.

### 3.2. DNA Methylation Changes for Wizened Buds and Their Relation with the Transcription of Various Genes

In addition to expression regulation, a change in the genome-wide DNA methylation status represents a control strategy during the plant transition from vegetative to reproductive growth [8,25]. Dynamic changes in DNA methylation during flower development have been detected in several species, which included apples [8], *Arabidopsis* [26], and pears [27]. In our study, WGBS was used to investigate global DNA methylation patterns in flower buds from the pear ‘Sucui 1’. The pattern of the pear ‘Sucui 1’ flower bud was similar to that of the roots of another pear species (*Pyrus betulaefolia*). However, the degree of methylation in all sequence contexts (^m^CG, ^m^CHG, and ^m^CHH) between root and flower bud differs [28]. Moreover, the total number of ^m^Cs and the methylation levels of the three sequence contexts between the two types of flower buds from ‘Sucui 1’ varied. This discrepancy may be the result of using different pear species and tissues in the two studies.

In the model plant *Arabidopsis*, CG hypermethylation is usually followed by local CHG and CHH hypomethylation in the developing flower buds [26]. However, in apples, the mean methylation level of the CG sequence context is the highest, followed closely by the CHG sequence. The methylation levels in the CHH context are the lowest in all types of flower buds [8]. Our analyses indicated that the CG context in TF DNA sequences was significantly hypermethylated in SM relative to CKM buds (Figure 3). Additionally, the hypermethylation of CHG and CHH at the chromosome level was accompanied by increased CG methylation (Figure 3). Our experiments indicated that the mobile adjustment of DNA methylation was crucial for flower development, despite different plant species showing varying trends for methylation changes.

Transcription factors respond to flower bud formation, flower opening, or senescence by modifying the DNA methylation states of their own gene sequences. In this study, there were 467 DMRs (including ^m^C, ^m^CG, ^m^CHG, and ^m^CHH) in the DNA sequences of TF genes in the two flower buds of *P. pyrifolia*. Furthermore, 207 TF genes displayed differential methylation level, including AP2/ERF, bHLH, MADS, MYB, and other family members, and their transcription levels were affected in SM.

For instance, sweet osmanthus (*Osmanthus fragrans*) ethylene-responsive (*OfERF*) TFs are strongly activated by DNA hypomethylation in senescent flowers [29]. Similarly, pear *PpERF011* is up regulated by DNA hypomethylation in SM (Figure 6 and Figure 8). On the contrary, *PpERF4* and *PpERF061* show the opposite trend (Figure 6 and Figure 8). Apple *bHLH* genes are strongly expressed in spur buds containing maximum flowering rate, which is related to hypomethylation levels among regions in the gene-body [8]. Here, pear *PpbHLH40* is expressed higher in SM and has a low DNA methylation level (Figure 6 and Figure 8). In contrast, *PpbHLH130* displays a lower expression level but has a hypermethylated DNA sequence (Figure 6 and Figure 8). However, bHLHs often interact with MYBs and form regulatory complexes to control floral transition [14,16]. Consequently, further research focusing on how *PpMYB308* and *PpbHLH40/PpbHLH130* combine and work together would be helpful in revealing the internal regulatory mechanisms of SM formation. *Arabidopsis* MADS-domain protein FLOWERING LOCUS C works as a chief floral repressor, which is methylated and down regulated in flower verbalization [30]. *PpMADS6* demonstrates a down regulation of expression and a hypermethylated condition in SM (Figure 6 and Figure 8). Our results indicate that the TFs in the buds of *P. pyrifolia*, as in other plants, regulate gene transcription by changing the DNA methylation levels of their own gene sequences, thereby participating in the flower development process. This could lead to an effective method of preventing flower bud development failure through influencing the expression levels of TFs by modulating their methylation status. One possible approach is to apply exogenous substances in orchards. This agricultural management practice offers the possibility of reducing the proportion of wizened buds in the future.

## 4. Conclusions

A comparison of SM and CKM revealed 467 DMRs and 207 DMGs of TFs. Among the DMGs, 162 TFs (containing 164 DMRs) displayed both expression differences and methylation level variations. These belonged to the AP2/ERF, bHLH, MADS, and MYB families, which commonly play roles in flower development. Combined with our bioinformatics and qPCR data, we hypothesized that TFs regulate their own expression to take part in the development of pear flower buds through altering DNA methylation states. Our experiments help us to recognize and understand the relationship between TF DNA methylation and flower bud development, and this will help to avoid flower development failure in pears.

## 5. Materials and Methods

### 5.1. Plant Material

The eight-year-old sand pear (*P. pyrifolia*) variety ‘Sucui 1’ was chosen as the experimental material. This variety was grafted onto *Pyrus calleryana* (a widely used rootstock) in 2014 and planted at Lishui Plant Science Base, Jiangsu Academy of Agricultural Sciences, Nanjing, Jiangsu Province, China (32°28′ N, 118°37′ E). Beginning on 1 May 2022, the lateral buds of the annual branches were observed every week. At the beginning of September, some buds lost external scales. After two weeks, the tips of these buds were grayish brown and wizened. Once approximately 50% of the buds were wizened, the annual branches were picked and taken to the laboratory. Then, the third buds from the branch above were classified as CKM or wizened SM (Supplementary Materials, Figure S1). Both types were independently snap-frozen in liquid nitrogen and then stored at −80 °C for further analysis.

### 5.2. RNA Sequencing and Data Analysis

The purified total RNA of pear flower buds was obtained using the TRIzol reagent (Invitrogen, Carlsbad, MA, USA) method. An RNA library was constructed using RNA that met the following criteria: RNA concentration > 100 ng·μL^−1^, RIN number > 7.0, OD_260/280_ > 1.8 and total content > 20 μg. Then, a transcriptomic library was prepared via an NEBNext^®^ Ultra^TM^ RNA Library Prep Kit (NEB, Ipswich, MA, USA), and 150 bp paired-end reads were generated through the Illumina NovaSeq 6000 platform (Genepioneer, Nanjing, China). CKM- and SM-based libraries were constructed using three biological replicates each.

FastQC (http://www.bioinformatics.babraham.ac.uk/projects/fastqc/, accessed on 6 March 2023) was used to test quality control indexes, and Trim fastp (v0.20.0) [31] was hired to filter joint sequences and low-quality sequences. HISAT (v2.1.0) [32] was chosen to align the filtered clean sequences to the reference pear genome [18] using default parameters. The obtained alignment was saved in a BAM file, and the fragments per kilo-base per million read values were calculated to represent the gene expression level. The gene read count was used as the input file, and R package ‘DESeq2’ (v4.2.0) [33] was applied to identify differentially expressed genes (DEGs), with the criteria |Log_2_ fold change| > 1 and false discovery rate (FDR) < 0.05. Finally, iTAK (v1.7a) software [34] was selected to identify TFs from the DEGs in the pear genome.

### 5.3. WGBS and Data Analysis

The genomic DNA from pear flower buds was extracted by the DNeasy Plant Mini Kit (Qiagen, Hilden, Germany) and depurated through MinElute PCR Cleanup (Qiagen). After ultrasonic treatment, mechanical disruption, terminal repair 3′ terminal A-base addition, and methylation ligand addition, 1 μg genomic DNA was processed via the EZ DNA Methylation Gold^TM^ kit (Zymo Research, Irvine, CA, USA), then converted into bisulfite. The whole-genome bisulfite sequencing (WGBS) library was constructed using the Illumina NovaSeq 6000 platform (Genepioneer, Nanjing, China) for two-end sequencing. The CKM and SM samples had three biological replicates each.

Trim fastp (v0.20.0) [31] was selected for quality filtering and splice removal from the original sequencing reads. Subsequently, Bismark (v0.22.3) and Bowtie2 (2.3.5.1) [35] were chose to compare the high-quality pruned read segments with the reference pear genome (https://www.ncbi.nlm.nih.gov/datasets/genome/GCA_007844245.1/, accessed on 6 March 2023) [18]. Then, the methylated cytosines in comparison readings were identified using Bismark (command—no_overlap). The screening conditions for ^5m^C detection were read coverage ≥ 4× and FDR < 0.05. MethylKit (v1.12.0) [36] was used to pick up differentially methylated regions (DMRs) in R package (v3.6.0), with the following parameters: span = 1000, FDR < 0.05, C > 0.15, CG > 0.20, CHG > 0.15, and CHH > 0.10.

### 5.4. Combined Transcriptome and Methylome Analysis

The DMR region was annotated from the WGBS analysis using Bedtools (v2.21.0) [37]. The Perl programming language was selected to obtain differentially methylated genes (DMGs) for further research. They formed the intersecting set of DEGs from the transcriptome and the DMRs at specified locations. Finally, the TF information identified by iTAK (v1.7a) software was used to determine the TF members containing DMGs [34].

### 5.5. Quantitative Real-Time PCR (qPCR)

The gene-specific primers for qPCR were designed through Primer Premier 5.0. Their sequences are shown in Table 5. In order to ensure primer specificity, PCR amplification, electrophoresis, and dissolution curves were tested for all the primers. qPCR was run on A LightCycle^®^ 480 II (Roche, Basel, Switzerland), and the reaction system followed the Genius 2 × SYBR Green Fast qPCR Mix (AB clone, Wuhan, China) instructions. *PbEF-1α* was selected as an internal control gene, and the relative expression levels of target genes were calculated using the 2^−ΔΔCT^ equation [38,39].

### 5.6. Statistical Analyses

The experimental data were run and analyzed on SPSS 26 (IBM, Armonk, New York, NY, USA), and the significant differences (** *p* < 0.01) in gene expression between CKM and SM were analyzed using Student’s *t*-tests. Their values are shown as the means ± standard errors from three biological replicates.

## Figures and Tables

**Table 1:** Gene family distributions of differential transcription factors (DTFs).

Transcription Factor Family *	The Number of DTFs	Up Regulated	Down Regulated
AP2/ERF-AP2	5	0	5
AP2/ERF-ERF	25	21	4
ARID	2	0	2
AUX/IAA	5	1	4
B3	9	5	4
B3-ARF	6	0	6
BES1	2	0	2
bHLH	25	13	12
bZIP	9	7	2
C2C2-CO-like	1	1	0
C2C2-Dof	3	1	2
C2C2-GATA	6	1	5
C2C2-YABBY	7	0	7
C2H2	19	9	10
C3H	2	1	1
GARP-G2-like	6	2	4
GNAT	3	3	0
GRAS	17	10	7
GRF	5	0	5
HB-BELL	3	0	3
HB-HD-ZIP	11	5	6
HB-KNOX	3	1	2
HB-other	1	1	0
HMG	4	0	4
HSF	5	4	1
IWS1	1	1	0
Jumonji	2	2	0
LIM	1	0	1
LOB	6	4	2
MADS-MIKC	2	1	1
mTERF	1	0	1
MYB	25	16	9
MYB-related	8	4	4
NAC	18	13	5
NF-YA	1	0	1
PHD	2	1	1
PLATZ	1	1	0
RWP-RK	2	2	0
SBP	2	0	2
SET	1	0	1
SNF2	2	0	2
SRS	2	0	2
SWI/SNF-BAF60b	2	0	2
TAZ	1	1	0
TCP	4	0	4
Tify	5	5	0
TRAF	3	2	1
Trihelix	2	1	1
TUB	2	0	2
WRKY	22	22	0
Zf-HD	9	0	9
Others	9	4	5
Total	320	166	154

* A list of abbreviations is shown in Supplementary Materials, Table S3.

**Table 2:** Compared analysis for the number of differential methylation or differential expression transcription factors in two flower buds of the pear variety ‘Sucui 1’.

Group	Methylation Type	Hypermethylation		Hypomethylation	
		Gene Region *	Promoter Region **	Gene Region *	Promoter Region **
CKM vs. SM	^m^C	9 (9)	2 (2)	7 (7)	3 (3)
	^m^CG	1 (1)	1 (1)	1 (1)	1 (1)
	^m^CHG	3 (3)	2 (2)	3 (3)	3 (3)
	^m^CHH	54 (53)	19 (18)	36 (36)	19 (19)

*: The numbers of intersections of differentially methylated gene regions and differentially expressed genes are shown in parentheses. **: The numbers of intersections of differentially methylated promoter regions and differentially expressed genes are shown in parentheses.

**Table 3:** Information on 10 differentially methylated transcription factors.

Gene Id	Regulated Type	Chromosome	Start	End	Width	Methylation Point Number	CKM Methylaion Level	SM Methylaion Level
GWHGAAYT001858	Hyper-Down	Chr01	15599546	15599577	32	15	0.09	0.28
GWHGAAYT035774	Hyper-Down	Chr03	15306247	15306341	95	20	0.03	0.13
GWHGAAYT039561	Hyper-Down	Chr04	22432346	22432441	96	19	0.11	0.24
GWHGAAYT009204	Hyper-Down	Chr11	30365284	30365413	130	8	0.06	0.20
GWHGAAYT018155	Hyper-Down	Chr14	15904276	15904336	61	8	0.13	0.32
GWHGAAYT019397	Hyper-Down	Chr14	24878942	24879068	127	10	0.04	0.19
GWHGAAYT028370	Hyper-Down	Chr17	3498720	3498755	36	6	0.04	0.25
GWHGAAYT034057	Hypo-Up	Chr02	25937363	25937466	104	13	0.26	0.13
GWHGAAYT003822	Hypo-Up	Chr10	17456977	17457036	60	6	0.21	0.09
GWHGAAYT020193	Hypo-Up	Chr15	4683725	4683873	149	34	0.26	0.13

**Table 4:** The correlations among the methylation, transcriptome, and qPCR results.

Gene Name	Gene ID	Gene Annotation	Fold Change (CKM vs. SM) *		
			Methylation	Transcriptome	qPCR
*PpZFP90*	GWHGAAYT028370	Zinc finger protein 90-like	6.88	−3.10	−1.57
*PpLHW*	GWHGAAYT035774	Transcription factor LHW-like	5.23	−2.39	−2.09
*PpZF-HD11*	GWHGAAYT019397	Zinc-finger homeodomain protein 11-like	4.84	−3.73	−2.44
*PpERF4*	GWHGAAYT009204	Ethylene-responsive transcription factor 4	3.29	−3.42	−2.74
*PpMADS6*	GWHGAAYT001858	MADS-box transcription factor 6-like	3.22	−2.31	−2.37
*PpERF061*	GWHGAAYT018155	Ethylene-responsive transcription factor ERF061	2.41	−2.76	−2.92
*PpbHLH40*	GWHGAAYT039561	Transcription factor bHLH140-like	2.12	−6.08	−2.84
*PpERF011*	GWHGAAYT020193	Ethylene-responsive transcription factor ERF011	−2.05	2.65	2.12
*PpbHLH130*	GWHGAAYT034057	Transcription factor bHLH130	−2.10	2.42	4.68
*PpMYB308*	GWHGAAYT003822	MYB-related protein 308-like	−2.41	1.85	5.22

* The correlation between the methylation and transcriptome is −0.78, and the correlation between the transcriptome and qPCR is 0.90.

**Table 5:** Primers used for qPCR in this study.

Primer Name	Forward Primer Sequence (5′-3′)	Reverse Primer Sequence (5′-3′)	Product Length (bp)
GWHGAAYT001858/*MADS*	TGAACAAAATCCTCGAGCGG	CGTTGAAGAGCCTCGTGTTTG	127
GWHGAAYT009204/*AP2/ERF*	CCGTCAACATCGCCAACA	GGGGGGTTTTGAGAGTGAGG	144
GWHGAAYT019397*/zf-HD*	CTCAGCCACGTCATCGCAA	CCTCCACCGGCATTATAGTCA	147
GWHGAAYT020193/*AP2/ERF*	TCACCGCCAAGAAAAGCA	TGTAGGAGCCGAGCCAAAT	110
GWHGAAYT028370/*C2H2*	ATCACGCTGGTTATGGATTACG	TGTGCCCGAACATCGCTCT	159
GWHGAAYT034057/*bHLH*	TTTTGAGATGCCTGCTATGGA	TGCCGTGTTTGTTTGCTTG	190
GWHGAAYT035774/*LHW*	GGAGTTGCGTGATATTGTGCC	AATCTTCGACTCTCCCGTTTGT	139
GWHGAAYT039561/*bHLH*	TCAGATATGGCTTCACCAGACC	TGAAGCAGCAGCACTAACGAA	241
GWHGAAYT003822/*MYB*	CCTGGAAGAACAGACAACGAGA	CAGAAGCGGCAGCAAAAGA	151
GWHGAAYT018155/*AP2/ERF*	ACGGGAAGGTTGTGAAGATGG	TGTAACAGGACGGCGGTGAG	147
GWHGAAYT023062/*PpActin*	AATGAACTTCGTGTTGCTCCTG	CACCTGAGTCCAGCACAATACC	196

## Data Availability

Data can available from https://www.ncbi.nlm.nih.gov/sra (search number from SRR24058259 to SRR24058270, accessed on 8 October 2024).

## Supplementary Materials

The following supporting information can be downloaded at: https://www.mdpi.com/article/10.3390/epigenomes8040040/s1, Figure S1: Morphology of wizened flower buds and normal flower buds from ‘Sucui 1’. Table S1: Information from the KEGG analysis of differential expressed genes in CKM vs. SM. Table S2: Information from the GO functional annotation of differential expression genes in CKM vs. SM. Table S3: A list of abbreviations. Table S4: Information regarding 10 differentially methylated transcription factors.
