# Supplementary material for: Transcription Factors Are Involved in Wizened Bud Occurrence During the Growing Season in the Pyrus pyrifolia Cultivar ‘Sucui 1’
Source: Epigenomes. 2024 Oct 25;8(4):40. doi: 10.3390/epigenomes8040040 (PMC11587157; doi:10.3390/epigenomes8040040)
Supplement: Supplementary file 1 [file epigenomes-08-00040-s001.zip › Supplementary Table S4 The replenish information of 10 differential methylation transcription factors (re).pdf]

Table S4. The replenish information of 10 differential methylation transcription factors.

Gene Id	CKM Depth	SM Depth	p value	Significant	Annotation	Gene start	Gene end	Gene chromosome
GWHGAAYT001858	23.27	11.8	2.46E-08	Hyper	Intron (2 of 6)	15596380	15603020	01
GWHGAAYT035774	11.75	10.85	1.35E-05	Hyper	Promoter (1-2kb)	15293303	15304553	03
GWHGAAYT039561	17.53	10.74	2.25E-04	Hyper	Promoter (<=1kb)	22431137	22431733	04
GWHGAAYT009204	12.63	11.5	4.60E-03	Hyper	Promoter (<=1kb)	30363672	30364793	11
GWHGAAYT018155	20.63	14.38	1.84E-04	Hyper	Promoter (1-2kb)	15900948	15902464	14
GWHGAAYT019397	19.9	22.1	7.99E-07	Hyper	Promoter (<=1kb)	24878306	24878695	14
GWHGAAYT028370	9	17.67	4.10E-04	Hyper	Downstream (<1kb)	3499579	3501198	17
GWHGAAYT034057	15.15	16.54	4.24E-04	Hypo	Promoter (<=1kb)	25934775	25936609	02
GWHGAAYT003822	16.83	32.83	5.26E-03	Hypo	Downstream (1-2kb)	17458564	17460100	10
GWHGAAYT020193	27.38	23.85	2.60E-12	Hypo	Promoter (1-2kb)	4685871	4686405	15

Gene Id	Gene length	Gene strand	Distance to TSS	CKM FPKM	SM FPKM	p value	q value	Regulated type
GWHGAAYT001858	6641	1	3166	18.38	7.95	1.11E-04	1.24E-03	down
GWHGAAYT035774	11251	2	-1694	13.98	5.84	4.29E-05	5.60E-04	down
GWHGAAYT039561	597	2	-613	2.22	0.36	4.90E-04	4.27E-03	down
GWHGAAYT009204	1122	2	-491	117.78	34.49	7.41E-03	3.68E-02	down
GWHGAAYT018155	1517	2	-1812	0.72	0.26	5.54E-03	2.96E-02	down
GWHGAAYT019397	390	2	-247	29.53	7.91	4.98E-05	6.38E-04	down
GWHGAAYT028370	1620	2	2443	1.67	0.54	4.05E-04	3.63-03	down
GWHGAAYT034057	1835	2	-754	1.37	3.31	5.75E-07	1.34E-05	up
GWHGAAYT003822	1537	2	3064	21.44	39.72	4.11E-06	7.46E-05	up
GWHGAAYT020193	535	1	-1998	17.81	47.27	2.08E-05	3.03E-04	up
