# Supplementary figures and images for: Transcription Factors Are Involved in Wizened Bud Occurrence During the Growing Season in the Pyrus pyrifolia Cultivar ‘Sucui 1’
Source: Epigenomes. 2024 Oct 25;8(4):40. doi: 10.3390/epigenomes8040040 (PMC11587157; doi:10.3390/epigenomes8040040)

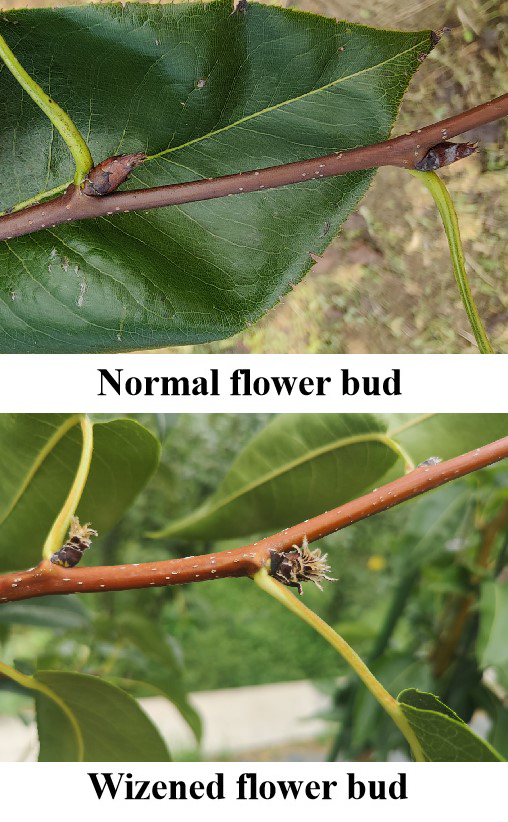

Supplement: Supplementary file 1 [file epigenomes-08-00040-s001.zip › Supplementary FigureS1 (re).tif]
